# VDR–SOX2 signaling promotes colorectal cancer stemness and malignancy in an acidic microenvironment

**DOI:** 10.1038/s41392-020-00230-7

**Published:** 2020-09-09

**Authors:** Pei-Shan Hu, Ting Li, Jin-Fei Lin, Miao-Zhen Qiu, De-Shen Wang, Ze-Xian Liu, Zhan-Hong Chen, Lu-Ping Yang, Xiao-Long Zhang, Qi Zhao, Yan-Xing Chen, Yun-Xin Lu, Qi-Nian Wu, Heng-Ying Pu, Zhao-Lei Zeng, Dan Xie, Huai-Qiang Ju, Hui-Yan Luo, Rui-Hua Xu

**Affiliations:** 1 Key Laboratory of Oncology in South China, Collaborative Innovation Center for Cancer Medicine, Sun Yat-sen University Cancer Center, 510060 Guangzhou, People’s Republic of China; 2 Precision Diagnosis and Treatment for Gastrointestinal Cancer, Chinese Academy of Medical Sciences, 510060 Guangzhou, People’s Republic of China; 3 Department of Medical Oncology and Guangdong Key Laboratory of Liver Disease, the Third Affiliated Hospital of Sun Yat-sen University, 510060 Guangzhou, People’s Republic of China

**Keywords:** Cancer stem cells, Gastrointestinal cancer, Epigenetics

## Abstract

The acidic tumor microenvironment provides an energy source driving malignant tumor progression. Adaptation of cells to an acidic environment leads to the emergence of cancer stem cells. The expression of the vitamin D receptor (VDR) is closely related to the initiation and development of colorectal carcinoma (CRC), but its regulatory mechanism in CRC stem cells is still unclear. Our study revealed that acidosis reduced VDR expression by downregulating peroxisome proliferator-activated receptor delta (PPARD) expression. Overexpression of VDR effectively suppressed the stemness and oxaliplatin resistance of cells in acidosis. The nuclear export signal in VDR was sensitive to acidosis, and VDR was exported from the nucleus. Chromatin immunoprecipitation (ChIP) and assay for transposase-accessible chromatin with high-throughput sequencing (ATAC-seq) analyses showed that VDR transcriptionally repressed SRY-box 2 (*SOX2*) by binding to the vitamin D response elements in the promoter of *SOX2*, impairing tumor growth and drug resistance. We demonstrated that a change in the acidic microenvironment combined with overexpression of VDR substantially restricted the occurrence and development of CRC in vivo. These findings reveal a new mechanism by which acidosis could affect the stemness of CRC cells by regulating the expression of SOX2 and show that abnormal VDR expression leads to ineffective activation of vitamin D signaling, resulting in a lack of efficacy of vitamin D in antineoplastic process.

## Introduction

Colorectal carcinoma (CRC) is a common malignancy whose morbidity and mortality rates rank it among the top five malignancies worldwide.^1^ Despite advances in the treatment of CRC, many patients show tumor progression with existing therapies.^2–4^ Genomic studies have shown that CRC has obvious heterogeneity and is influenced by a variety of epigenetic modifications that are closely related to the existence and heterogeneity of CRC stem cells. Notably, the tumor microenvironment can regulate the functions and fates of tumor cells. Strategies targeting CRC stem cells are urgently needed to prevent CRC recurrence and improve the prognosis of CRC patients.

The tumor microenvironment has an important role in determining the cancer stem cell (CSC) phenotype.^5,6^ Low oxygen and low pH are two physicochemical characteristics of the tumor microenvironment. These characteristics lead to a series of changes related to cancer cell biological phenotypes, including an induced CSC phenotype and a metabolic reprogramming phenotype.^7^ In addition, low oxygen and low pH change the core cell metabolic phenotype, providing the basic conditions required for cancer cells to optimize their metabolism.^8,9^ Our recent studies have investigated the roles of metabolic microenvironment reprogramming in promoting gastrointestinal cancer progression.^10–13^ However, the effects of acidic stress on cancer and the underlying mechanisms need further study. Thus, we sought to investigate the effects of the acidic tumor microenvironment on CRC stem cells.

Vitamin D has long been considered to have potential applications in cancer therapy. The active form of vitamin D (1α,25-(OH)-2-D3) can inhibit tumor growth.^14^ Vitamin D regulates the transcription of target genes by binding with the vitamin D receptor (VDR),^15^ which has a transcriptional regulatory role through binding of its DNA-binding domains with vitamin D response elements (VDREs) on target genes.^16^ Studies have shown that the VDR expression level is related to the degree of differentiation of cancer cells, and we have previously found that an acidic environment can suppress the vitamin D signaling pathway to promote the CSC phenotype among glioma cells.^17^ These findings suggest that the expression of VDR is closely related to the initiation and development of cancer, but the VDR regulatory mechanism in CRC stem cells is still unclear. Therefore, clarification of the effects of vitamin D and VDR on the stemness of CRC stem cells and elucidation of the interaction between CRC stem cells and the acidic tumor microenvironment are important.

In this study, we found that the acidic tumor microenvironment can reduce VDR expression via PPARD and prevent the accumulation of VDR in the nucleus. This relieves the inhibitory effect of VDR on the SOX2 promoter, thereby promoting SOX2 expression and leading to tumor growth and drug resistance.

## Results

### Acidosis inhibits VDR expression, which is negatively correlated with malignant CRC

**Fig. 1:**
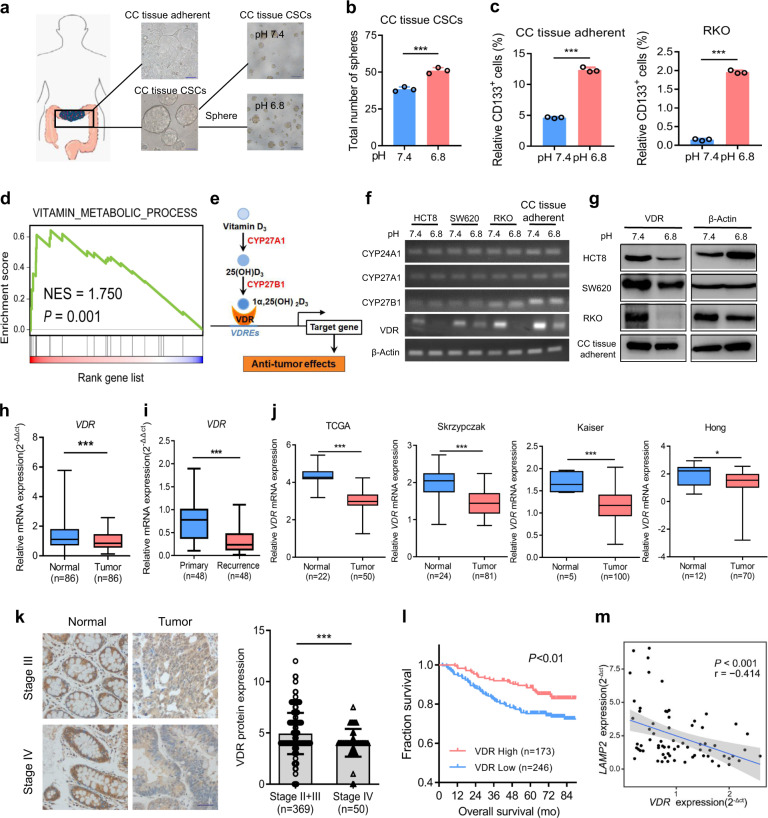
VDR is negatively correlated with the acidic microenvironment-associated CRC malignancy. **a**, **b** Schematic representation of the method for isolation of primary CRC cells and CRC stem cells. Representative images (**a**) and quantified data for tumor spheres (with diameters larger than 50 µm) formed by CC tissue CSCs cultured under pH 7.4 and 6.8 (**b**). Student’s *t*-test. **c** Flow cytometric analysis of CD133-positive CRC cells (CC tissue adherent and RKO cells) cultured under pH 7.4 and 6.8. Student’s *t*-test. **d** Enriched signaling pathways from the gene ontology (GO) database in RKO cells cultured under pH 7.4 and 6.8. **e** Schematic diagram illustrating the key factors (CYP27A1, CYP27B1, VDR, and CYP24A1) involved in anabolism and catabolism of 1α,25(OH)_2_D_3_ and the regulation of target genes. CYP27A1 cytochrome P450 family 27 subfamily A member 1, CYP27B1 cytochrome P450 family 27 subfamily B member 1, VDR vitamin D receptor, CYP24A1 cytochrome P450 family 24 subfamily A member 1. **f**, **g** qPCR (**f**) and immunoblotting (**g**) of CYP24A1, CYP27A1, CYP27B1, and VDR in CRC cells (HCT8, SW620, RKO, and CC tissue-adherent cells) cultured under pH 7.4 and 6.8. **h**, **i** qPCR of *VDR* in 86 CRC samples and paired adjacent normal samples (**h**) and in 48 primary CRC tissue and 48 recurrent CRC tissue samples from our hospital (SYSUCC; **i**). Student’s *t*-test. **j** qPCR data for *VDR* in normal tissue samples and CRC tissue samples from TCGA and other databases (obtained through Oncomine; https://www.oncomine.com). **k**, **l** Representative immunohistochemical images (left) and quantified data (right) for VDR (**k**) in 419 CRC tissue and adjacent normal tissue samples from representative patients with different stages of CRC at our hospital (SYSUCC). The overall survival of patients with low and high VDR expression (**l**) are shown. Student’s *t*-test, Kaplan–Meier method and the log-rank test were used. **m** Correlation between *LAMP2* expression and *VDR* expression in 86 CRC samples, as described in Fig. 1h. The Pearson correlation coefficient (r) and *P* value are shown. Three independent experiments were performed to obtain the data in **b**, **c** and **f**, **g**. The data are shown as the mean ± SD; *P < 0.05, **P < 0.01, and ***P < 0.001

To investigate the effect of the acidic tumor microenvironment on the stemness of CRC cells, we first isolated and identified RKO stem-like cells (RKO-SLCs) and primary CRC cells (CC tissue-adherent cells), CRC stem cells (CC tissue CSCs) from tissue samples of CRC patients (Supplementary Fig. S1a–c). The cells were cultured in medium with different pH values to evaluate the optimum conditions for cell self-renewal (Supplementary Fig. S1d). The number of tumor spheres was maximized under acidic conditions with a pH of 6.8 (Fig. 1a, b; Supplementary Fig. S1d). Compared with a pH of 7.4, a pH of 6.8 significantly increased the percentage of CD133-positive cells and promoted the expression of the stemness markers prominin 1 (CD133), POU class 5 homeobox 1 (OCT4) and SOX2 in non-stem CRC cells (Fig. 1c; Supplementary S1e, f). Thus, the acidic tumor microenvironment could induce and maintain the CSC phenotypes of CRC cells.

To determine the important pathways involved in the regulation of the CSC phenotype in the acidic microenvironment, we analyzed RKO cells cultured under pH 7.4 and 6.8 by RNA sequencing and performed signaling pathway enrichment analysis. We found that the metabolic process of fat-soluble vitamins was significantly changed by the acidic conditions (Fig. 1d). Vitamin D is an important fat-soluble vitamin, and the vitamin D signaling pathway is known to be involved in the regulation of cancer cell differentiation.^14^ Therefore, we tested the expression of several proteins that have key roles in the vitamin D signaling pathway (Fig. 1e) under pH 7.4 and 6.8, and found that VDR expression in each cell line was significantly decreased under acidosis (Fig. 1f, g). The mRNA expression of *VDR* differed markedly between CRC tissues and paired adjacent tissues, while the expression of CYP24A1, CYP27A1, and CYP27B1 did not differ significantly (Fig. 1h; Supplementary Fig. S1g). Furthermore, VDR expression in recurrent CRC tissues was significantly reduced (Fig. 1i). In the Cancer Genome Atlas (TCGA) and the Skrzypczak, Kaiser, and Hong databases, *VDR* expression in CRC tissue was lower than that in normal tissue (Fig. 1j), and VDR showed the lowest expression in stage-IV CRC tissues (Fig. 1k). We also examined the relationship between VDR expression and prognosis, and the results showed that patients with low VDR expression had short survival times (Fig. 1l). The expression of lysosomal-associated membrane protein 2 (*LAMP2*) is positively correlated with tumor microenvironment acidity,^18^ and we found that *VDR* expression and *LAMP2* expression in CRC were negatively correlated (Fig. 1m). These results suggest that the acidic tumor microenvironment can inhibit VDR expression, which is closely related to the degree of malignancy and the recurrence of CRC.

### VDR impairs stemness and malignancy in CRC

**Fig. 2:**
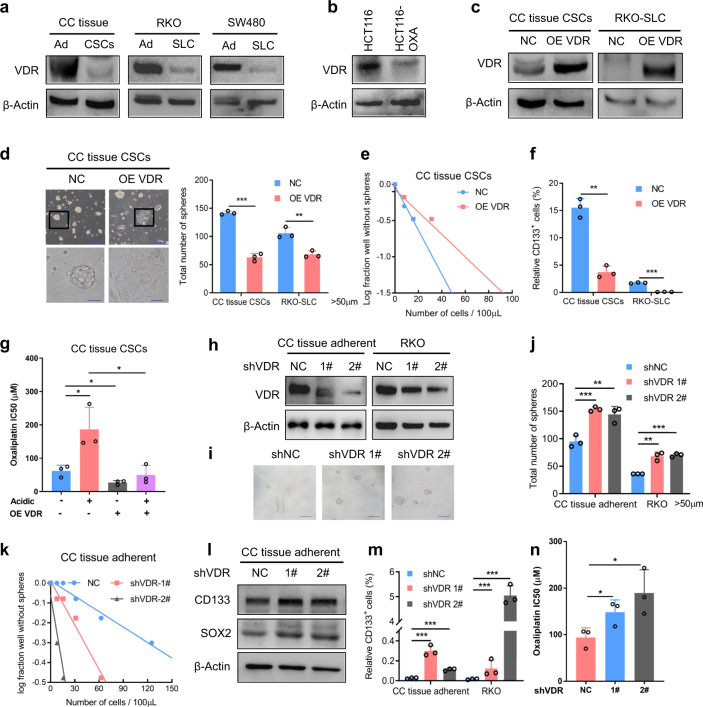
VDR impairs the CSC phenotype and drug resistance in CRC. **a** Immunoblotting of VDR in CC tissue adherent (Ad) cells and CSCs, RKO (Ad) cells and RKO-SLCs, and SW480 (Ad) cells and SW480-SLCs. CSCs, cancer stem cells; SLCs, stem-like cells. **b** Immunoblotting of VDR in HCT116 and oxaliplatin-resistant HCT116 (HCT116-OXA) cells. **c** Immunoblotting of VDR in CC tissue CSCs and RKO-SLCs infected with negative control and VDR overexpression lentiviruses. **d** Representative images (left) and quantified data (right) for tumor spheres (with diameters larger than 50 µm) formed by control and VDR-overexpressing CC tissue CSCs. Scale bars: upper, 200 μm; lower, 50 μm. Student’s *t*-test. **e** Limiting dilution assay of control and VDR-overexpressing CC tissue CSCs. Wells not containing tumor spheres (with diameters larger than 50 μm) for each cell plating density were used for the calculations after 1–2 weeks. **f** The percentages of CD133-positive cells among control and VDR-overexpressing CC tissue CSCs and RKO-SLCs were evaluated using flow cytometric analysis. Student’s *t*-test. **g** IC50 of oxaliplatin in control and VDR-overexpressing CC tissue CSCs treated with oxaliplatin at pH 7.4 and 6.8. Student’s *t*-test. **h**–**j** Immunoblotting of VDR in CC tissue-adherent cells and RKO cells treated with control or VDR-targeting shRNA (**h**). Representative images (**i**) and quantified data (**j**) for tumor spheres (with diameters larger than 50 µm) formed by CC tissue adherent and RKO cells treated with control or VDR-targeting shRNA. Scale bars: 200 μm. Student’s *t*-test. **k** Limiting dilution assay of CC tissue-adherent cells treated with control or VDR-targeting shRNA. **l** Immunoblotting of the stemness markers CD133 and SOX2 in CC tissue-adherent cells treated with control or VDR-targeting shRNA. **m** The percentages of CD133-positive cells among CC tissue adherent and RKO cells treated with control or VDR-targeting shRNA were evaluated using flow cytometric analysis. Student’s *t*-test. **n** IC50 of oxaliplatin in CC tissue-adherent cells treated with control or VDR-targeting shRNA and with oxaliplatin. Student’s *t*-test. Three independent experiments were performed to obtain the data in **d**–**g**, **j**, **k**, **m**, and **n**. The data are shown as the mean ± SD; *P < 0.05, **P < 0.01, and ***P < 0.001

To investigate the effects of VDR on the phenotypes of CRC stem cells, we first confirmed that VDR expression was lower in CRC stem cells than in non-stem cancer cells (Fig. 2a). VDR expression was also confirmed to be lower in CRC cell lines than in the normal colonic epithelial cell line (Supplementary Fig. S1h). In addition, VDR expression was decreased in oxaliplatin-resistant HCT116 cells (Fig. 2b). Therefore, we overexpressed VDR in CRC stem cells with low VDR expression (Fig. 2c). Microscopic imaging showed that the tumor spheres adhered to the bottoms of the plates and that the cellular differentiation occurred in the tumor spheres (Fig. 2d). VDR overexpression significantly reduced the sizes and numbers of tumor spheres formed by CRC stem cells (Fig. 2d, e; Supplementary Fig. S1i, j) and significantly reduced the percentage of CD133-positive cells (Fig. 2f). These results indicated that VDR overexpression inhibited the self-renewal of CRC stem cells and induced cell differentiation. In addition, VDR overexpression increased the sensitivity of CRC stem cells to oxaliplatin and partially attenuated the acidic tumor microenvironment-mediated promotion of drug resistance (Fig. 2g). Next, we observed that knockdown of VDR (Fig. 2h) could elevate the self-renewal ability of CRC cells (Fig. 2i–k; Supplementary Fig. S1k, l), promoting the expression of stemness markers (Fig. 2l; Supplementary Fig. S1m, n). Moreover, knockdown of VDR increased the percentage of CD133-positive cells (Fig. 2m) and strengthened oxaliplatin resistance (Fig. 2n). All these findings demonstrate that VDR inhibits the CSC phenotype and enhances the sensitivity of CRC stem cells to drugs in the acidic tumor microenvironment.

### The acidic tumor microenvironment regulates the stemness and drug resistance of CRC through the VDR-SOX2 axis

**Fig. 3:**
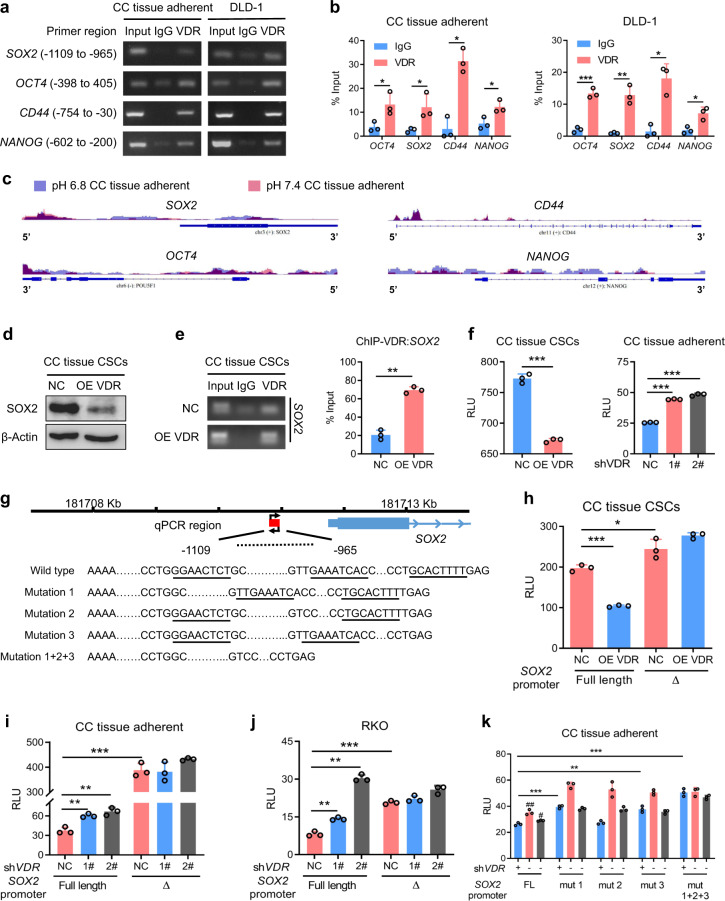
VDR downregulates SOX2 expression by inhibiting the transcriptional activity of the SOX2 promoter. **a**, **b** ChIP was performed to assess VDR binding to the promoters of *SOX2*, *OCT4*, *CD44*, and *NANOG* in CC tissue adherent and DLD1 cells (**a**). A polyclonal anti-VDR antibody or a mouse IgG antibody was used. The immunoprecipitated DNA was quantified by qPCR (**b**). Student’s *t*-test. **c** ATAC-seq enrichment from 2500 bp upstream of the TSSs throughout the whole ranges of the *SOX2*, *OCT4*, *CD44*, and *NANOG* genes in CC tissue-adherent cells cultured under pH 7.4 (red) and pH 6.8 (blue). **d** Immunoblotting of SOX2 in control and VDR-overexpressing CC tissue CSCs. **e** The extent of VDR binding to the SOX2 promoter in control and VDR-overexpressing CC tissue CSCs was measured by ChIP assay (left). The immunoprecipitated DNA was quantified by qPCR (right). A polyclonal anti-VDR antibody or a mouse IgG antibody was used. Student’s *t*-test. **f** The transcriptional regulatory activity of VDR on the promoter of SOX2 in control and VDR-overexpressing CC tissue CSCs (left) and CC tissue-adherent cells treated with control or VDR-targeting shRNA (right) was measured by dual-luciferase reporter assay. RLU, relative luciferase unit. **g** Schematic representation of the SOX2 promoter containing three VDREs. The mutation strategy of the promoter is shown. **h**–**j** The transcriptional regulatory activity of VDR on the full-length SOX2 promoter and a mutant promoter with deletion of sites −1109 to −965 in control and VDR-overexpressing CC tissue CSCs (**h**) and in CC tissue adherent and RKO cells treated with control or VDR-targeting shRNA (**i**–**j**) was measured by dual-luciferase reporter assay. △, SOX2 promoter mutant with deletion of sites −1109 to −965. **k** The transcriptional regulatory activity of VDR on the full-length SOX2 promoter and on a triple VDRE–mutated SOX2 promoter (as described in **g**) in CC tissue-adherent cells treated with control or VDR-targeting shRNA was measured by dual-luciferase reporter assay. FL, full length. Three independent experiments were performed to obtain the data in **b**, **e**, **f**, and **h**–**k**. The data are shown as the mean ± SD; *P < 0.05, **P < 0.01, and ***P < 0.001

In order to clarify how VDR affects the CSC phenotype and drug resistance in CRC, we used chromatin immunoprecipitation (ChIP) to analyze the regulatory effects of VDR on the transcription of stemness markers and found that VDR could bind to the promoter regions of *SOX2*, *OCT4*, *CD44*, and *NANOG* (Fig. 3a, b). Then, we investigated the chromatin accessibility of the stemness genes in cultured CRC cells under either acidic or alkaline culture condition by analyzing assay transposase-accessible chromatin with high-throughput sequencing (ATAC-seq) signals from upstream of transcription start sites (TSSs) throughout the whole ranges of the *SOX2*, *OCT4*, *CD44*, and *NANOG* genes. The open chromatin in *SOX2* preferentially occurred upstream of the TSS in acidic conditions (Fig. 3c), which may reflect the possibility of VDR binding at the promoter region of *SOX2*. We verified that SOX2 protein expression was decreased by overexpression of VDR (Fig. 3d) and found that the binding effect was enhanced in cells overexpressing VDR (Fig. 3e). Overexpression of VDR inhibited the transcriptional activity of the *SOX2* promoter, while knockdown of VDR promoted it (Fig. 3f). We further deleted the binding sequence in the *SOX2* promoter region (Fig. 3g) and found that the inhibitory effect of VDR on the *SOX2* promoter was attenuated (Fig. 3h–j). Through bioinformatics analysis, we found three VDREs in the *SOX2* promoter (Fig. 3g). We deleted the three VDRE sites and detected the effects of VDR on the transcriptional activity of the *SOX2* promoter. Mutation 1 and mutation 3 significantly weakened the inhibitory effect of VDR (Fig. 3k). These results suggest that VDR may downregulate SOX2 expression by inhibiting *SOX2* promoter activity.

**Fig. 4:**
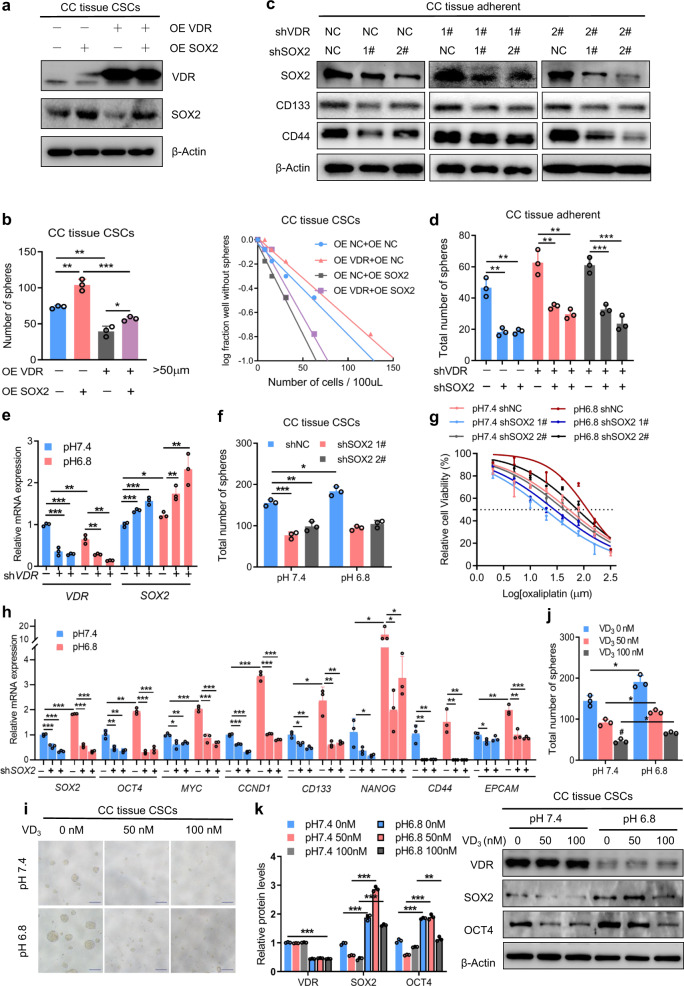
SOX2 overexpression reverses the VDR-mediated inhibition of stemness, and the vitamin D-VDR signaling pathway affects the stemness of CRC in acidic environments. **a** Immunoblotting of VDR and SOX2 in control and VDR-overexpressing CC tissue CSCs with or without SOX2 overexpression. **b** Tumor sphere formation assays (left) and limiting dilution assays (right) of control and VDR-overexpressing CC tissue CSCs with or without SOX2 overexpression. Student’s *t*-test. **c** Immunoblotting of SOX2, CD133, and CD44 in control and VDR-knockdown CC tissue-adherent cells treated with control or SOX2-targeting shRNA. **d** Tumor sphere formation assays of control and VDR-knockdown CC tissue-adherent cells treated with control or SOX2-targeting shRNA. Student’s *t*-test. **e** qPCR of *VDR* and *SOX2* in control and VDR-knockdown CC tissue CSCs cultured under pH 7.4 and 6.8. Student’s *t*-test. **f** Tumor sphere formation assays of control and SOX2-knockdown CC tissue CSCs under pH 7.4 and 6.8. Student’s *t*-test. **g** Cell viability of control and SOX2-knockdown CC tissue CSCs with oxaliplatin treatment under pH 7.4 and 6.8. The IC50 is shown as a dotted line. **h** qPCR of *SOX2*, *OCT4*, *MYC*, *CCND1*, *CD133*, *NANOG*, *CD44*, and *EPCAM* in control and SOX2-knockdown CC tissue CSCs cultured under pH 7.4 and 6.8. Student’s *t*-test. **i**, **j** Representative images (**i**) and quantified data (**j**) for tumor spheres formed by CC tissue CSCs treated with 0, 50, or 100 nM of the active form of vitamin D (1α,25-(OH)-2-D3, VD_3_) under pH 7.4 and 6.8. Scale bars: 200 μm. Student’s *t*-test. **k** Immunoblots (right) and quantified levels (left) of VDR, SOX2, and OCT4 in CC tissue CSCs treated with 0, 50, or 100 nM of the active form of VD_3_ under pH 7.4 and 6.8. Student’s *t*-test. Three independent experiments were performed to obtain the data in **b**, **d**–**h**, **j**, and **k**. The data are shown as the mean ± SD; *P < 0.05, **P < 0.01, and ***P < 0.001

To confirm that VDR suppresses the malignant phenotype of CRC cells by downregulating SOX2 expression, we overexpressed SOX2 in CRC stem cells overexpressing VDR (Fig. 4a; Supplementary Fig. S2a). The self-renewal ability of CRC stem cells increased upon SOX2 overexpression (Fig. 4b; Supplementary Fig. S2b). Knockdown of SOX2 in CRC cells with low VDR levels reduced the expression of CD133 and CD44 (Fig. 4c; Supplementary Fig. S2c) and decreased self-renewal ability (Fig. 4d). We also analyzed the *SOX2* mRNA in CRC stem cells with and without VDR expression in both acidic and alkaline pHs. Results showed that *SOX2* mRNA was increased in cells without VDR expression in acidic pH (Fig. 4e). Consistent with the previous results, knockdown of SOX2 in the acidic tumor microenvironment weakened the self-renewal ability of CRC stem cells (Fig. 4f; Supplementary Fig. S2d) and strengthened the sensitivity of the cells to oxaliplatin (Fig. 4g). And we detected the mRNA expression of stem cell gene networks at pH 6.8 when silencing SOX2. Results showed that the stem cell gene, *OCT4*, *MYC*, and*CCND1*, which are also target genes of SOX2, were decrease when silencing SOX2. And other stem cell genes were also downregulated when silencing SOX2 (Fig. 4h). Moreover, we found that the active form of vitamin D could reverse the acidic environment-mediated promotion of self-renewal and CD133, SOX2, and OCT4 expression in CRC stem cells (Fig. 4i, j; Supplementary Figs. S2e and 4k), suggesting that the acidic microenvironment affects the stemness of CRC cells through the vitamin D-VDR signaling pathway. Together, our findings demonstrate that downregulation of VDR in the acidic tumor microenvironment relieves the transcriptional inhibition of SOX2, resulting in increased SOX2 expression. These changes promote the stemness and drug resistance of CRC cells.

### The acidic microenvironment inhibits VDR aggregation in the nucleus and suppresses VDR expression through PPARD

**Fig. 5:**
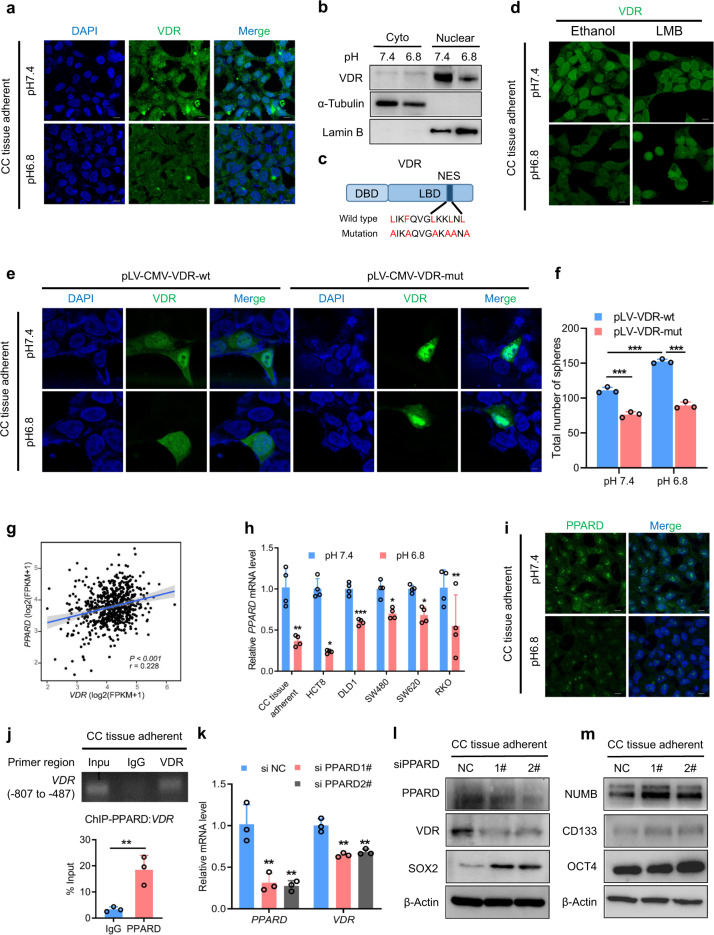
The acidic tumor microenvironment inhibits VDR aggregation in the nucleus and suppresses the expression of VDR through PPARD. **a** Immunofluorescence staining of VDR (green) and DAPI (blue) in CC tissue-adherent cells under pH 7.4 and 6.8. Scale bars: 10 μm. **b** Immunoblotting of VDR in the cytoplasm and nucleus in CC tissue-adherent cells cultured under pH 7.4 and 6.8. α-Tubulin is mainly expressed in the cytoplasm. Lamin B is a fibrous protein that exhibits a structural function and performs transcriptional regulation in the cell nucleus. **c** Schematic representation of the NES site in the LBD domain of VDR and the mutated amino acids in NES mutant. NES, nuclear export signal. LBD, ligand-binding domain. DBD, DNA-binding domain. **d** Immunofluorescence staining of VDR (green) in CC tissue-adherent cells treated with ethanol or LMB for 1 h at 37 °C under pH 7.4 and 6.8. LMB, leptomycin B (a nuclear export protein inhibitor). Scale bars: 10 μm. **e** Immunofluorescence staining of VDR (green) and DAPI (blue) in CC tissue-adherent cells transfected with pLV-CMV-VDR or pLV-CMV-VDR-mut (the wild-type or mutated VDR shown in **c**, respectively). Scale bars: 5 μm. **f** Tumor sphere formation assays of CC tissue CSCs transfected with pLV-CMV-VDR or pLV-CMV-VDR-mut under pH 7.4 and 6.8. Student’s *t*-test. **g** Correlation of PPARD expression with VDR expression in CRC samples from TCGA. **h** qPCR of *PPARD* in CC tissue adherent, HCT8, DLD1, SW480, SW620, and RKO CRCs cultured under pH 7.4 and 6.8. Student’s *t*-test. **i** Immunofluorescence staining of PPARD (green) and DAPI (blue) in CC tissue-adherent cells under pH 7.4 and 6.8. Scale bars: 10 μm. **j** ChIP was used to assess PPARD binding to the promoters of VDR in CC tissue-adherent cells (upper). A polyclonal anti-PPARD antibody or a mouse IgG antibody was used. The immunoprecipitated DNA was quantified by qPCR (lower). Student’s *t*-test. **k** qPCR of *PPARD* and *VDR* in CC tissue-adherent cells transfected with siRNA for PPARD. Student’s *t*-test. **l**, **m** Immunoblotting of PPARD, VDR, SOX2, and the stemness markers NUMB, CD133, and OCT4 in CC tissue-adherent cells transfected with siRNA for PPARD. NUMB is an endocytic adaptor protein that has a crucial role in asymmetrical cell division. Three independent experiments were performed to obtain the data in **f**, **h**, **j**, and **k**. The data are shown as the mean ± SD; *P < 0.05, **P < 0.01, and ***P < 0.001

VDR regulates target genes in the nucleus. Thus, we detected the subcellular localization of VDR in acidic environments. We found that VDR expression was decreased and that VDR protein accumulation in the nucleus was reduced under acidic conditions (Fig. 5a, b). Further analyses showed that the intracellular pH value was decreased under acidic conditions (Supplementary Fig. S2f) and that VDR contained a nuclear export signal (NES) (Fig. 5c). Nuclear export protein inhibitor leptomycin B (LMB) binds to nuclear export receptor (chromosome region maintenance 1, CRM1) and inhibits the binding of other export substrates.^19^ We used LMB to treat CRC cells under pH 7.4 and 6.8. LMB prevented the nuclear export of the VDR protein in pH 6.8 culture medium (Fig. 5d), indicating that the nuclear export of the VDR protein in acidic environments is dependent on CRM1. To confirm that the nuclear export of VDR requires NES, we transfected CRC cells with wild-type and NES site-mutant (Fig. 5c) plasmids and found that the VDR NES mutation prevented VDR nuclear export in an acidic environment (Fig. 5e). To investigate whether the expression of nuclear VDR make cells insensitive to pH-driven reprogramming, we transfected CC tissue CSCs with wild-type and NES site-mutant plasmids, and subjected them to acidic conditions. The results showed that the NES mutation of VDR inhibited the self-renewal of colorectal cancer stem cells under acidic conditions (Fig. 5f; Supplementary Fig. S2g, h). This finding indicates that the nuclear export of VDR in the acidic tumor microenvironment is mediated by the NES and is dependent on CRM1.

Using the Qiagen database, we predicted that peroxisome proliferator-activated receptor (PPAR) family members, which are closely related to tumor occurrence, were associated with the VDR promoter. We first analyzed the correlations among PPARA, PPARD, PPARG, and VDR in CRC data in the TCGA database (Fig. 5g; Supplementary Fig. S3a, b). PPARA and PPARD expression was positively correlated with VDR expression (Supplementary Fig. S3b; Fig. 5g). We also found that *PPARD* mRNA and protein levels in CRC cells were significantly decreased under acidic conditions (Fig. 5h, i), while *PPARA* and *PPARG* levels were not significantly changed (Supplementary Fig. S3c). Surprisingly, PPARD could bind to the promoter of *VDR* (Fig. 5j), and knockdown of PPARD decreased the mRNA and protein expression of VDR and upregulated the expression of stemness markers (Fig. 5k–m; Supplementary Fig. S3d). These results suggest that the acidic tumor microenvironment inhibits the expression of VDR through PPARD, inducing nuclear export of the VDR protein and inhibiting the transcriptional regulatory function of VDR.

### Normalization of the acidic tumor microenvironment and induction of VDR expression restrain the initiation and development of CRC

**Fig. 6:**
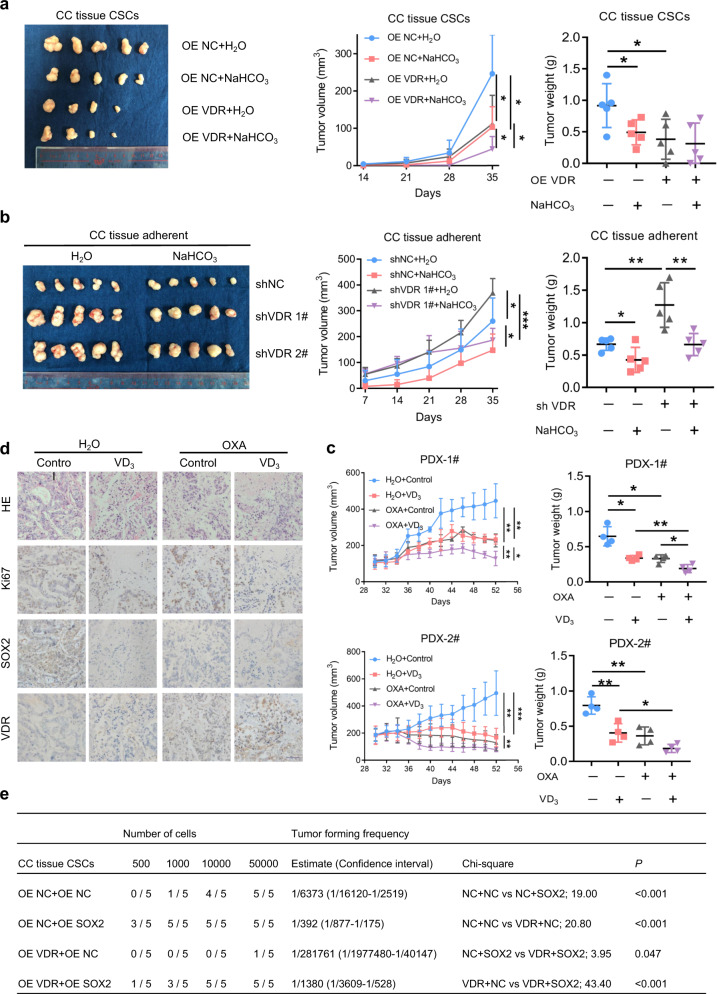
Normalization of the acidic tumor microenvironment and induction of VDR expression restrain the initiation and development of CRC. **a**, **b** Tumor images (left), tumor volumes (middle) and tumor weights (right) of mice (*n* = 5) subcutaneously injected with control or VDR-overexpressing CC tissue CSCs (**a**) or with control or VDR-knockdown CC tissue-adherent cells (**b**) and subsequently treated with water or 200 mM sodium bicarbonate (NaHCO_3_). Tumor weight was measured after 35 days. **c**, **d** Tumor volumes (left) and tumor weights (right) of mice (*n* = 4) with or without oxaliplatin and VD_3_ treatment in two PDX models (**c**). The immunohistochemical images show VDR and SOX2 expression in a representative case (**d**). Scale bars: 50 μm. **e** Tumor formation frequencies in mice (*n* = 5) subcutaneously injected with control or VDR-overexpressing CC tissue CSCs with or without SOX2 overexpression. The cells were limiting diluted. The frequencies were calculated by software from http://bioinf.wehi.edu.au/software/elda/. *P* < 0.05 was used as the significance threshold for comparisons between the different groups. The data are shown as the mean ± SD; *P < 0.05, **P < 0.01, and ***P < 0.001

To determine whether CRC growth can be attenuated by modification of the acidic tumor microenvironment and VDR expression, we injected CRC cells into nude mice and gave the mice water or a sodium bicarbonate (NaHCO_3_) solution.^20,21^ We found that NaHCO_3_ treatment after VDR overexpression significantly inhibited SOX2 expression (Supplementary Fig. S3e, f) and tumor development (Fig. 6a) and that NaHCO_3_ treatment also effectively attenuated SOX2 expression and tumor formation after VDR knockdown (Supplementary Fig. S3e, g; Fig. 6b). These findings suggest that CRC development can be inhibited by decreasing the acidity of the tumor microenvironment and inducing VDR expression. We further found that a combination of vitamin D signaling activation and oxaliplatin treatment could inhibit the tumor growth of the patient-derived xenografts (PDXs) (Fig. 6c). VDR expression was upregulated and SOX2 expression was downregulated in the PDXs (Fig. 6d; Supplementary Fig. S3h). These results provide a new theoretical basis for the clinical treatment of CRC.

**Fig. 7:**
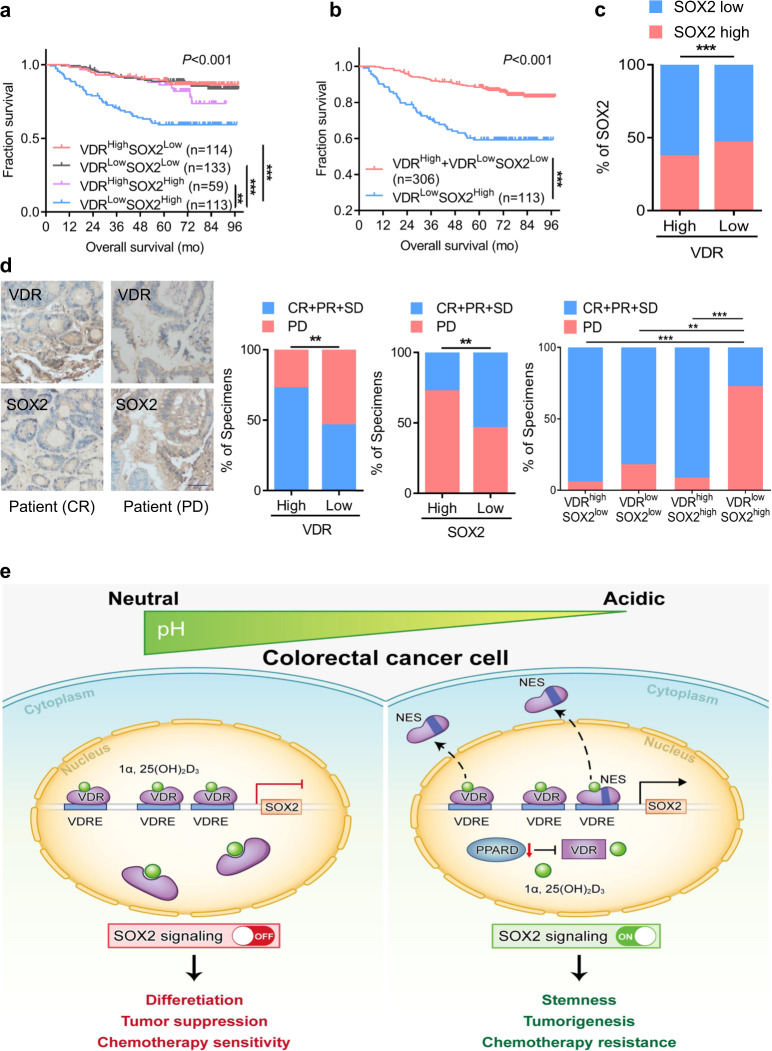
VDR expression is negatively correlated with SOX2 expression and has the potential to be used for clinical prognosis prediction. **a**, **b** Overall survival of patients according to the expression of VDR and SOX2 in 419 CRC tissue samples, as described in Fig. 1k. The Kaplan–Meier method and the log-rank test were used. **c** Correlation between VDR expression and SOX2 expression in 253 stages III–IV CRC tissue samples from our hospital (SYSUCC). The data are presented as the percentage of total samples. Pearson’s Chi correlation analysis was used. **d** The percentages of specimens with low/high VDR and SOX2 expression relative to the response to FOLFOX or XELOX chemotherapy were analyzed (middle and right). The results for two representative cases are shown (left). Pearson chi square test. PD progressive disease, CR complete response, PR partial response and SD stable disease. Scale bars: 50 μm. **e** Schematic illustration of VDR-SOX2 signaling in CRC cells under acidosis. There are three VDREs in the promoter region of SOX2. The transcription factor VDR transcriptionally represses SOX2 by binding to the VDRE, reducing SOX2 expression. The acidic tumor microenvironment upregulates SOX2 in a VDR-dependent manner and facilitates the stemness and malignancy of CRC

Finally, we verified the influences of VDR and SOX2 expression on the tumorigenic ability of CRC stem cells in vivo. The results of the limiting dilution experiment in vivo showed that overexpression of VDR significantly inhibited tumor occurrence, whereas overexpression of SOX2 attenuated the repressive effect of VDR overexpression (Fig. 6e). Overall survival (OS) was significantly different among patients with high/low expression levels of VDR and SOX2 (*P* < 0.001). Patients with both low expression of VDR and high expression of SOX2, which were unfavorable for survival, had the worst prognoses (*P* < 0.001) (Fig. 7a, b). The expression levels of VDR and SOX2 were negatively correlated among stage-III and stage-IV specimens (Fig. 7c). Furthermore, we evaluated the expression of VDR and SOX2 in samples from 65 patients with advanced CRC treated with the FOLFOX or XELOX regimens. Only 27.69% of patients with high VDR expression in their primary tumors showed resistance to chemotherapy (progressive disease, PD), whereas 72.31% of patients with high SOX2 expression showed resistance to chemotherapy. The group of patients with both low VDR expression and high SOX2 expression had the highest proportion of chemotherapy resistance (Fig. 7d). These results indicate that both low VDR expression and high SOX2 expression predict resistance to oxaliplatin-based chemotherapy.

## Discussion

Acidity is a basic characteristic of the tumor microenvironment, and provides an energy source driving the malignant progression of tumors. Adaptation of cells to acidic environments leads to the emergence of tumor cells with increased aggression, proliferation, and drug resistance.^22,23^ Thus, acidic microenvironments are favorable for the survival and growth of tumor cells but unfavorable for the survival and growth of normal cells. Acidic environments directly regulate tumor cell invasion by affecting immune cell function, cell clone evolution, and drug resistance.^24^ Researchers have long assumed that acidic environments are associated with hypoxia. However, interestingly, acidic regions are not confined to hypoxic regions in the tumor–stroma interface but rather overlap with regions where cells proliferate and invade. The expression of matrix metalloproteinases is increased in these regions, and the basement membrane is degraded.^25,26^ Transcriptome studies have shown that tumor-related stressors, such as hypoxia, nutritional deficiencies, and lactic acid-mediated acidification, can regulate gene expression at the transcriptional and posttranscriptional levels in vitro.^20,27,28^ For example, low extracellular pH levels lead to increased histone deacetylation, which affects the expression of certain stress response genes and promotes the normalization of intracellular pH, primarily through enhancement of the release of protons by the monocarboxylate transporter (MCT).^29,30^ Smad5 has been shown to positively respond to changes in intracellular pH (pHi) and to shuttle from the nucleus to cytoplasm.^19^ Moreover, an acidic environment can activate eEF2K and enhance the phosphorylation of eEF2. Five histidine residues in eEF2K have been found to have crucial roles in the activation of eEF2K under acidic conditions.^31^ Here, we discovered that the NES in VDR is sensitive to acidic conditions and that VDR is exported from the nucleus. These results indicate that the acidic tumor microenvironment can affect VDR-mediated transcriptional regulation of target genes by changing the subcellular localization of VDR. Specifically, the acidic environment may regulate the activity of VDR by influencing leucine residues in it. The acidic environment would change the cellular proteome and cellular metabolism to a large scope; we discovered that VDR could attenuate the acidic tumor microenvironment-mediated promotion of CSC phenotype. Acidic environment affected SOX2 expression through VDR, and it is known that SOX2 affects a wide variety of proteins related to stem cell pluripotency. But the correlation between other changes of cellular proteome and VDR need further investigation.

According to previously reported statistics, high levels of circulating 25(OH)D significantly reduce CRC risk in women but not in men. The optimal concentration of 25(OH)D for CRC risk reduction is 75–100 nmol/l, which is higher than the current Institute of Medicine (IOM) recommendations.^32^ This finding suggests that low VDR expression may reduce the risk of CRC by increasing the levels of vitamin D. Notably, vitamin D3 supplementation has not been found to significantly reduce the incidence of aggressive tumors. Similarly, compared with placebo treatment, vitamin D treatment does not reduce overall cancer mortality or the incidence of breast cancer, prostate cancer, or CRC.^33^ Such clinical findings show that vitamin D is not very effective in reducing the incidence of cancer or improving the prognoses of cancer patients. We hypothesized that cancer-related abnormalities in VDR, the key factor mediating the function of vitamin D, lead to ineffective activation of the vitamin D signaling pathway, causing vitamin D to be ineffective as a cancer treatment. In our study, we found that overexpression of VDR could effectively suppress the CSC phenotype, decrease invasion, and increase sensitivity to oxaliplatin in CRC cells in acidic environments.

Another study has shown that VDR is expressed in the stroma in human pancreatic cancer. The VDR ligand calcipotriol can significantly decrease the levels of inflammation and fibrosis markers in inflamed pancreas tissue and the tumor stroma. As the major transcriptional regulatory factor of pancreatic stellate cells (PSCs), VDR can restore the quiescent state of cells; thus, compared with chemotherapy alone, VDR induce tumor stromal remodeling, increase gemcitabine levels in tumor tissue, reduce tumor volumes, and improve survival rates.^34^ We also demonstrated that VDR can significantly suppress the growth of tumors and that modification of the acidic tumor microenvironment combined with VDR overexpression substantially restricts the occurrence and development of CRC in vivo.

SOX2 is a transcription factor with a high-mobility group domain and sequence-specific DNA-binding activity.^35^ This transcription factor is not only necessary for embryonic stem cells but also a key factor for induced pluripotent stem cells.^36^ The expression of SOX2 is increased in many cancers and is associated with poor prognosis.^37,38^ SOX2 has roles in maintaining tumor-initiating cells, and in determining the self-renewal ability and tumorigenic potential of various types of cancer cells.^39,40^ SOX2 can also regulate other transcription factors; for example, this molecule can interact with OCT3/4 to regulate the transcription of NANOG and other pluripotent-related genes, such as FGF4, UTF1, and LEFTY1.^35^ However, other transcription factors can in turn regulate SOX2. Previous reports have shown that CDK1 binds SOX2 and regulates its phosphorylation, nuclear transport, and transcriptional activity, thus promoting tumorigenesis. CDK1 is therefore a new SOX2 regulator in tumor cells.^41^ In addition, SIX2 is a transcription factor with homologous domains. Six2 directly binds the srr2 enhancer of SOX2 to promote the expression of SOX2 in breast cancer, indicating that a SIX2/SOX2 axis is necessary for effective metastatic cloning and highlighting the critical role of the stemness factor SOX2 in tumor growth at metastatic sites.^42^ In a previous study, miR-638 was found to inhibit the luciferase activity of a reporter gene connected to the 3′ UTR of SOX2 in CRC.^43^ SOX2 has also been confirmed to be a target of miR-200c, as miR-200c inhibits SOX2 expression and blocks PI3K–AKT pathway activity. Moreover, miR-200c and SOX2 mutually control their expression levels through feedback loops.^44^ However, the roles of transcription factors in the regulation of SOX2 in CRC remain unclear. Our study shows that there are three VDREs in the promoter region of *SOX2*. We found that the transcription factor VDR transcriptionally represses *SOX2* by binding to the VDREs, consequently reducing SOX2 expression. Overexpression of SOX2 can markedly facilitate CRC growth in vivo. Furthermore, the acidic tumor microenvironment alters SOX2 in a VDR-dependent manner (Fig. 7e). These findings reveal a new mechanism through which the acidic tumor microenvironment can affect the CSC phenotype of CRC cells by regulating the expression of the pluripotent transcription factor SOX2. We suppose that there is antagonism between those factors and VDR, but it needs further investigation. In addition, some factors might bind to the upstream of TSSs in *SOX2* gene, and most of them activate the expression of *SOX2*. Under acidic condition, VDR was exported from the nucleus into cytoplasm, the inhibiting effect of VDR on SOX2 expression was relieved, and those factors could bind to the open chromatin at upstream of TSSs in *SOX2* gene. We suppose that there is antagonism between those factors and VDR, and this is an exciting future area of investigation.

## Materials and methods

A detailed description of the methods can be found in the Supplementary materials.

### Cells and specimens

Human CRC cell lines and immortalized colon epithelial cells were obtained from the American Type Culture Collection (Manassas, VA, USA) and cultured as recommended. All cells tested negative for mycoplasma contamination and were authenticated by short tandem repeat (STR) fingerprinting before use. All CRC specimens were obtained with written informed consent from all patients. The clinical characteristics of the samples are summarized in Supplementary Tables S1 and  S2.

### Isolation of primary CRC cells and CSCs

Primary CRC cells and CSCs were isolated from fresh CRC samples obtained from surgery. The tumor tissues were minced and digested in collagenase I (Gibco, Grand Island, NY, USA) and 0.05% trypsin (Gibco, Grand Island, NY, USA) at 37 °C for 1 h. Then, the digested tissue was filtered using a 40 μm cell strainer. Tissue-adherent CC cells were cultured in RPMI 1640, and CC tissue CSCs were cultured in DMEM/F12 stem medium (DMEM/F12 supplemented with 20 ng/ml basic fibroblast growth factor, 20 ng/ml epidermal growth factor, 10 μg/ml heparin, and 2% B27).

### Acidic culture conditions

HEPES (25 mM) and PIPES (25 mM) (Sigma-Aldrich, St. Louis, MO, USA) were added to RPMI 1640 medium containing 10% FBS and 1% penicillin–streptomycin or to DMEM/F12 stem medium, and the pH value was titrated using 1 M HCl or 1 M NaOH. The medium was incubated for 24 h and then retitrated.

### ChIP

ChIP was performed as described previously.^45^ A MAGnify Chromatin Immunoprecipitation System (Invitrogen, Carlsbad, CA, USA) was used according to the manufacturer’s instructions. A total of 5–10 × 10^6^ cultured cells were used per test, and 4 μg of anti-VDR antibody or 1 μg of isotype-control antibody (mouse IgG) was used per test. PCR and real-time quantitative PCR (qPCR) were used to verify the binding ability between VDR and the promoters of target genes. The primers used are listed in the Supplementary Methods. The data were calculated as the percentage of the input. Three independent experiments were repeated.

### ATAC-seq

A total of 5 × 10^5^ tissue-adherent CC cells cultured under pH 7.4 or 6.8 were processed according to a previously published protocol,^46^ and 150 bp paired-end sequencing was performed on Illumina Xten to yield an average of 97 M reads/sample.

### Tumor development and NaHCO_3_ treatment

Subcutaneous xenograft models were established as described previously.^17^ Briefly, 1 × 10^4^ CC tissue CSCs or tissue-adherent CC cells were injected subcutaneously into nude mice in 0.1 ml of Matrigel and PBS (1:4). After 1 week, 200 mM NaHCO_3_ or water was provided to the mice and remained available throughout the course of the experiment.^20^ The tumor volumes of the mice in each group (*n* = 5) were estimated each week using the formula *V* = *ab*^2^/2 (*V*, volume; *a*, length; *b*, width). After 35 days, the tumor tissues were dissected, and the weights were measured. The animal protocol was approved by the Animal Ethics Committee of Sun Yat-Sen University.

### Statistics

Student’s *t*-test was used for statistical analysis. The data are shown as the mean ± standard deviation and were analyzed by SPSS 20.0 software, and a *P* < 0.05 was considered to indicate statistical significance. Pearson correlation coefficients were used to analyze the expression correlations of different genes.

### Study approval

Informed consent was obtained from patients before surgery. The study was approved by the Medical Ethics Committee of Sun Yat-Sen University Cancer Center. The animal protocol was approved by the Animal Ethics Committee of Sun Yat-Sen University.

## Supplementary information

Supplementary Materials

## Data Availability

The original RNA sequencing data and ATAC-seq data have been uploaded to the Genome Sequence Archive (GSA; http://gsa.big.ac.cn/) and are accessible under the GSA numbers CRA001942 (RNA sequencing data) and CRA002255 (ATAC-seq data).
