# Supplementary material for: VDR–SOX2 signaling promotes colorectal cancer stemness and malignancy in an acidic microenvironment
Source: Signal Transduct Target Ther. 2020 Sep 9;5:183. doi: 10.1038/s41392-020-00230-7 (PMC7479104; doi:10.1038/s41392-020-00230-7)
Supplement: Supplementary file 1 — Supplementary Materials [file 41392_2020_230_MOESM1_ESM.docx]

**Supplementary Materials for**

**VDR-SOX2 Signaling Promotes Colorectal Cancer Stemness and Malignancy in an Acidic Microenvironment**

Pei-Shan Hu^1,2#^, Ting Li^1#^, Jin-Fei Lin^1#^, Miao-Zhen Qiu^1^, De-Shen Wang^1^, Ze-Xian Liu^1^, Zhan-Hong Chen^1,3^, Lu-Ping Yang^1^, Xiao-Long Zhang^1^, Qi Zhao^1^, Yan-Xing Chen^1^, Yun-Xin Lu^1^, Qi-Nian Wu^1^, Heng-Ying Pu^1^, Zhao-Lei Zeng^1^, Dan Xie^1^, Huai-Qiang Ju^1,2^, Hui-Yan Luo^1,2*^, Rui-Hua Xu^1,2*^

^1^Key Laboratory of Oncology in South China, Collaborative Innovation Center for Cancer Medicine, Sun Yat-sen University Cancer Center, Guangzhou 510060, P. R. China

^2^Precision Diagnosis and Treatment for Gastrointestinal Cancer, Chinese Academy of Medical Sciences, Guangzhou 510060, P. R. China

^3^Department of Medical Oncology and Guangdong Key Laboratory of Liver Disease, the Third Affiliated Hospital of Sun Yat-sen University, Guangzhou 510060, P. R. China

Correspondence to: [xurh@sysucc.org.cn](mailto:xurh@sysucc.org.cn) or [luohy@sysucc.org.cn](mailto:luohy@sysucc.org.cn)

^#^ These authors contributed equally to this work: Pei-Shan Hu, Ting Li, and Jin-Fei Lin

**This PDF file includes:**

Materials and Methods

Figures. S1 to S3

Tables S1 to S2

Materials and Methods

**Reagent and antibody**

| Reagent or Resource | Source | Identifier |
| --- | --- | --- |
| Antibody |  |  |
| Anti-CD133 antibody | abcam | ab19898 |
| Anti-Oct4 antibody | abcam | ab181557 |
| Anti-SOX2 antibody | abcam | ab97959 |
| Anti-Vitamin D Receptor antibody - ChIP Grade | abcam | ab3508 |
| Anti-β-actin | sigma | A5441 |
| CDX2 antibody | CST | 12306 |
| Keratin 20 antibody | CST | 13063 |
| Keratin 7 antibody | CST | 4465 |
| Anti-EpCAM antibody [E144] | abcam | ab32392 |
| Anti-CD44 antibody [EPR1013Y] | abcam | ab51037 |
| PPARD antibody | absin | abs102023 |
| Anti-Vitamin D Receptor antibody - ChIP Grade | abcam | ab3508 |
| Goat anti-Mouse IgG (H+L) Cross-Adsorbed Secondary Antibody, Alexa Fluor 594 | invitrogen | R37121 |
| Reagents |  |  |
| EGF | Peprotech | AF-100-15 |
| FGF-basic | Peprotech | 100-18B |
| heparin | Sigma | H3149 |
| PIPES | Sigma | RES0703P-A701X |
| HEPES | Sigma | H4034 |
| PIPES | Sigma | P1851 |
| Falcon® 40µm Cell Strainer | Corning | 352340 |
| B-27™ Supplement (50X), minus vitamin A | Gibco | 12587010 |
| StemPro™ Accutase™ Cell Dissociation Reagent | Gibco | A1110501 |
| CD133/2(293C3)-PE | Miltenyi biotec | 130-090-853 |
| mouse IgG2b isotype control antibodies | Miltenyi biotec | 130-092-215 |
| FcR blocking reagent | Miltenyi biotec | 130-059-901 |
| Natural mouse laminin | Gibco | 23017015 |
| Collagenase, Type I, powder | Gibco | 17100017 |
| StemPro™ Accutase™ Cell Dissociation Reagent | Gibco | A1110501 |
| ViaFect™ Transfection Reagent | promega | E4981 |
| BCECF AM | Beyotime | S1006 |
| Leptomycin B | Beyotime | S1726 |

**Cells and cell culture**

The human CRC cell lines (RKO, DLD1, SW480, SW620, HCT8, HCT15 and HCT116) and immortalized colon epithelial cells (NCM460) were obtained from the American Type Culture Collection (Manassas, VA, USA) and cultured as recommended. CC tissue adherent cells and CC tissue cancer stem cells were isolated from CRC tumor tissues, and cultured in RPMI 1640 supplemented with 10% FBS and penicillin/streptomycin (P/S) or DMEM/F12 supplemented with 20 ng/ml basic fibroblast growth factor (bFGF; Peprotech, Rocky Hill, NJ, USA), 20 ng/ ml epidermal growth factor (EGF; Peprotech, Rocky Hill, NJ, USA), 10 μg/ml heparin (Sigma-Aldrich, St Louis, MO, USA), and 2% B27 (Life Science), named DMEM/F12 stem medium. All cells tested negative for mycoplasma contamination, and were authenticated by STR fingerprinting before use.

**Immunoblotting and immunohistochemical (IHC) analysis**

Immunoblotting and IHC analysis were conducted as previously reported[1-3]. Anti-β-Actin antibody was used as a loading control. The IHC stained sections were reviewed and scored independently by two pathologists. A final score was then calculated.

**RNA isolation and qPCR analysis**

Total RNA was isolated with TRIzol reagent and 1 μg of total RNA was reverse-transcribed to cDNA in a volume of 20 μl with the Prime Script RT Master Mix Kit (TaKaRa, Tokyo, Japan) and served as the template for real-time PCR using GoTaq qPCR Master Mix (Promega, Madison, WI, USA) according to the manufacturer’s instructions. Data were analyzed and normalized to the β-Actin data. Primer sequences for qRT-PCR and ChIP assays are listed below:

| Name | Forward | Reverse |
| --- | --- | --- |
| Primer sequences for qRT-PCR | |  |
| NANOG | 5’-CCTCACACGGAGACTGTCTCTCC-3’ | 5’-CACCTGTTTGTAGCTGAGGTTCA-3’ |
| EPCAM | 5’-CGCAGCTCAGGAAGAATGTG-3’ | 5’-CATTTGGCAGCCAGCTTTG-3’ |
| CD44 | 5’-CGCAGATCGATTTGAATATAACC-3’ | 5’-CTATGAACCCATACCTGCAGG-3’ |
| LGR5 | 5’-GAGTTACGTCTTGCGGGAAAC-3’ | 5’-CCAGACGCAGGGATTGAAG-3’ |
| CYP27A1 | 5’-GTTCACCACGGAAGGACAC-3’ | 5’-TGTAGCAAATAGCTTCCAAGGC-3’ |
| CYP27B1 | 5’-GTTCGGACTGGAAGGCATC-3’ | 5’-GCCTCTGAGCAAATGCAAAC-3’ |
| VDR | 5’-CCTCTTCAGACATGATGGACTC-3’ | 5’-GTGAGGTCTCTGAATCCTGG-3’ |
| CYP24A1 | 5’-CCCAAAGGAACAGTGCTCATG-3’ | 5’-CTAATCGGCGACCAATGCAC-3’ |
| CD133 | 5’-TGAACTGAGGCAGCTTCCACCC-3’ | 5’-CGACAGTCGTGGTTTGGCGTT-3’ |
| SOX2 | 5’-TCAGGAGTTGTCAAGGCAGAGAAGA-3’ | 5’-TGCCGCCGCCGATGATTGT-3’ |
| OCT4 | 5’-TTGGGCTCGAGAAGGATGTGGT-3’ | 5’-ACGGAGACAGGGGGAAAGGCTT-3’ |
| PPARD | 5’-GGGTGCAAGGGCTTCTTC-3’ | 5’-GATAGCGTTGTGTGACATGCC-3’ |
| PPARA | 5’-AAGAGTAGCTTGGAGCTCGG-3’ | 5’-TGAAAGCGTGTCCGTGATGA-3’ |
| PPARG | 5’-GCCGTGGCCGCAGAAA-3’ | 5’-GGGAGTGGTCTTCCATTACGG-3’ |
| β-ACTIN | 5’-TGGATCAGCAAGCAGGAGTA-3’ | 5’-TCGGCCACATTGTGAACTTT-3’ |
| Primer sequences for ChIP assay | |  |
| SOX2-chip1 | 5’-CGCATTGAGCGCCTACCTAT-3’ | 5’-GATTTCCATTGTGGTGGCCG-3’ |
| SOX2-chip2 | 5’-AAAATGCCAGGGCTGGTTCT-3’ | 5’-CTCAAAAGTGCAGGCGATGG-3’. |
| SOX2-chip3 | 5’-GTTGAAATCACCCTCCCCCA-3’ | 5’-CATGCCTCCCCGTAAGAAGG-3’ |
| SOX2-chip4 | 5’-GGGGCAGACAGTAGAAGCAT-3’ | 5’-TTGGTAAGCTGCCATCTCCC-3’. |
| SOX2-chip5 | 5’-TTACCAAGGCCTGCTGGTTC-3’ | 5’-GTGTGTCATTGTTCTCCCGC-3’ |
| SOX2-chip6 | 5’-ATACGAGTTGGACAGCCGC-3’ | 5’-AGCAACAGGTCACACCACAC-3’. |
| SOX2-chip7 | 5’-AAATACGAGTTGGACAGCCGC-3’ | 5’-CTCTGCCTTGACAACTCCTGA-3’ |
| CD133-chip1 | 5’-CGATTAAAAGAGGCGGGACC-3’ | 5’-ATCTGCTGGGGTCCCATAAAC-3’. |
| CD133-chip2 | 5’-GGACCCCAGCAGATGTTCAA-3’ | 5’-ACAGTAGGACATGGCAAGGTT-3’ |
| CD133-chip3 | 5’-ACCTTGCCATGTCCTACTGT-3’ | 5’-GCACCTTCAGGATGGTGTCT-3’. |
| CD133-chip4 | 5’-TGTAGCTTGTGCATCCATCCT-3’ | 5’-CCTCGGAGTCTTCACCTTGC-3’ |
| CD133-chip5 | 5’-GCAAGGTGAAGACTCCGAGG-3’ | 5’-TAGTGGCTGAAGCGGTTGAA-3’. |
| CD133-chip6 | 5’-GGCACCTCTACAGGAAATGGA-3’ | 5’-GCTACTCACCGTGCACCC-3’ |
| CD133-chip7 | 5’-GGTCTGGCGAGCTAAGGGA-3’ | 5’-CCACTCCTCACTGTACACCCC-3’. |
| OCT4-chip1 | 5’-CCACCCTGATCACCCAGTTG-3’ | 5’-AGGTTGTGATTGATTCAGGATGT-3’ |
| OCT4-chip2 | 5’-TCGCTTGGACTAAAACAAAGTCAC-3’ | 5’-AGTCTAATGTGGCAAGGCCC-3’. |
| OCT4-chip3 | 5’-CTTCAGGGCCTTGCCACATTA-3’ | 5’-AGGGTAAAGGAGGGAAGGAGATT-3’ |
| OCT4-chip4 | 5’-TAAATAGAGGCAGCAGGGGTG-3’ | 5’-AGCCAGCGGCTATACAAAGT-3’. |
| OCT4-chip5 | 5’-TAGCACTTCTGTCATGCTGGA-3’ | 5’-ACTCTCCCCAGCTTGCTTTGA-3’ |
| Lgr5-chip1 | 5’-GATTGTGCGGAAACCGGAGT-3’ | 5’-TTGGAGAGAAGCCCTCGTTG-3’. |
| Lgr5-chip2 | 5’-GTCTGGCGAGGGCTATTTGT-3’ | 5’-TCCTATCTCTTGCGGGGTGA-3’. |
| Lgr5-chip3 | 5’-CAAAAGCGAGCCAGACAGTG-3’ | 5’-CGCCTGGAAGATTCAGTGCT-3’ |
| Lgr5-chip4 | 5’-TGGGCGCGCAATTCGG-3’ | 5’-CCTCAGCAACACACCAGACC-3’. |
| BMI1-chip1 | 5’-GTGAGGGCAAATAACCGATTTGA-3’ | 5’-AAAAGGACATCCAGCTCGCA-3’ |
| BMI1-chip2 | 5’-CACTTTAAAAACGTGTGAAAGGGTT-3’ | 5’-AGGAGATCGCATCGTTTCCTC-3’. |
| BMI1-chip3 | 5’-TGTTGTTTCCGCGAGCC-3’ | 5’-ACGTGCTCCCCTCATTCCTTA-3’. |
| BMI1-chip4 | 5’-GTGTGGCGCTGTGGAGAAAT-3’ | 5’-CATTGTCTCGCCCCGATCT-3’ |
| BMI1-chip5 | 5’-CTCCCAGCCCCGCAGAATAA-3’ | 5’-CCTCGCCTCCTACGTACCC-3’. |
| CD44-chip1 | 5’-CACTTCCCCTGTGAAGACTG-3’ | 5’-TTATCAGTGGCTGTGGCAAGA-3’ |
| CD44-chip2 | 5’-TTCTGTGTAACTCACCAGGCA-3’ | 5’-CACAACCATCCACCATCCTCT-3’. |
| CD44-chip3 | 5’-AACAGATATGTGGGTGGATGGG-3’ | 5’-CAAGCCAGTGTGAATGGGCA-3’. |
| CD44-chip4 | 5’-TGGGTTAGCTGAGCCAAATG-3’ | 5’-TCAGGACAGAGGATGACCGAA-3’ |
| CD44-chip5 | 5’-ACGGTTCGGTCATCCTCTGT-3’ | 5’-GCGTGCCACCAAAACTTGTC-3’. |
| ABCG2-chip1 | 5’-AACAGTCGTTAACGGCCAGG-3’ | 5’-ATTCATGATGCGCCCAAACA-3’. |
| ABCG2-chip2 | 5’-ATGGGCCTTTAAGGGTCTTGA-3’ | 5’-CCGATGGTGTGGAAAGGCTA-3’. |
| ABCG2-chip3 | 5’-CCATCGGAATATTGCACAGAAGT-3’ | 5’-TGGGCTGATCAGTACCTCGT-3’ |
| ABCG2-chip4 | 5’-GTCGCAGGGCGCTTATCG-3’ | 5’-CCCGGAACCTTTTGAGTGGG-3’. |
| NANOG-chip1 | 5’-CAGGGTTTCACCATGTTGG-3’ | 5’-CTTACTACATTCTTCGCCAAGG-3’. |
| NANOG-chip2 | 5’-GCTTGTGTGGGAGCAAAG-3’ | 5’-GGAGGTGTTTTCCAAAGGAAC-3’. |
| NANOG-chip3 | 5’-GACGTAGTCCCGCTCTGTTG-3’ | 5’-GAACCTGAAGACAAACCCAGC-3’ |
| NANOG-chip4 | 5’-GCGTTGACCCAACTTTATGG-3’ | 5’-CGGCTTTATGAGTATCCCG-3’. |
| ALDH1A1-chip1 | 5’-GAGCGCCACTCTCAAGTTATG-3’ | 5’-GTAAATATTTTCAGCCTCTGGGC-3’. |
| ALDH1A1-chip2 | 5’-CCTTAGTGGCCAGAGCAGC-3’ | 5’-GCACTTGGCTTTATTTGTTCC-3’. |
| ALDH1A1-chip3 | 5’-GGAACAAATAAAGCCAAGTGC-3’ | 5’-CACAGAAGAGAATTCCTTTCGC-3’ |
| ALDH1A1-chip4 | 5’-CCAAGTTTGTTGCCATTGG-3’ | 5’-GGCTCTCTCTTGATTTGCAAG-3’. |
| EPHB2-chip1 | 5’-CGAGCTCCTGATGAAGTCTCC-3’ | 5’-CTGTGGAATGGACCTGTCTG-3’. |
| EPCAM-chip1 | 5’-CGGAGTTTCGCTCTTGTTG-3’ | 5’-GGTATTTAGGCCAGGTACGGTG-3’. |
| EPCAM-chip2 | 5’-GGGTTTCACCATGTTGGC-3’ | 5’-GCCTTCGGAAAGAAAATATAGG-3’ |
| CD166-chip1 | 5’-GACGGACTTGGCTTTTAATCTG-3’ | 5’-GATATTTGCTTTTCCCGGG-3’. |

**In vitro cell migration assay**

The standard procedure was performed as previously described[4]. Briefly, CRC cells or control cells (2×10^5^) were harvested, suspended and transferred to the upper chamber. Cells on the lower surface of the membrane were stained and counted in at least three random view fields.

**Cell viability assay**

CRC cells (5 × 10^3^ cells/well) were seeded in 96-well plates overnight and then exposed to oxaliplatin (0.02 μM, 2 μM, 5 μM, 10 μM, 20 μM) for 24 h or 48 h, and cell viability was determined using MTS assays as previously described[5, 6].

**Immunofluorescence staining analysis**

Cells were harvested and cultured in chamber slides overnight and fixed. Then blocked for nonspecific binding and incubated with the indicated antibody at 4°C overnight (VDR or SOX2 was diluted at 1:1000). Then the cells were incubated with anti-rabbit IgG (H+L). Nuclei were visualized with 4',6-diamidino-2-phenylindole (DAPI). Images were acquired on a Zeiss Axio Observer Z1 fluorescence microscope. For each channel, all images were acquired with the same settings.

**RNA-sequencing**

RNA degradation and contamination was monitored on 1% agarose gels. RNA purity was checked using the NanoPhotometer® spectrophotometer (IMPLEN, CA, USA). RNA integrity was assessed using the RNA Nano 6000 Assay Kit of the Bioanalyzer 2100 system (Agilent Technologies, CA, USA). A total amount of 1 μg RNA per sample was used as input material for the RNA sample preparations. Sequencing libraries were generated using NEBNext® UltraTM RNA Library Prep Kit for Illumina® (NEB, USA) following manufacturer’s recommendations and index codes were added to attribute sequences to each sample. The clustering of the index-coded samples was performed on a cBot Cluster Generation System using TruSeq PE Cluster Kit v3-cBot-HS (Illumia) according to the manufacturer’s instructions. After cluster generation, the library preparations were sequenced on an Illumina Nova platform and 150 bp paired-end reads were generated.

References

1. Ju HQ, Lu YX, Chen DL*, et al.* Redox Regulation of Stem-like Cells Though the CD44v-xCT Axis in Colorectal Cancer: Mechanisms and Therapeutic Implications. Theranostics 2016;6(8):1160-75.

2. Lu YX, Ju HQ, Liu ZX*, et al.* ME1 Regulates NADPH Homeostasis to Promote Gastric Cancer Growth and Metastasis. Cancer Res 2018;78(8):1972-1985.

3. Ju HQ, Ying H, Tian T*, et al.* Mutant Kras- and p16-regulated NOX4 activation overcomes metabolic checkpoints in development of pancreatic ductal adenocarcinoma. Nat Commun 2017;8:14437.

4. Chen DL, Lu YX, Zhang JX*, et al.* Long non-coding RNA UICLM promotes colorectal cancer liver metastasis by acting as a ceRNA for microRNA-215 to regulate ZEB2 expression. Theranostics 2017;7(19):4836-4849.

5. Ju HQ, Lu YX, Wu QN*, et al.* Disrupting G6PD-mediated Redox homeostasis enhances chemosensitivity in colorectal cancer. Oncogene 2017;36(45):6282-6292.

6. Li S, Zhuang Z, Wu T*, et al.* Nicotinamide nucleotide transhydrogenase-mediated redox homeostasis promotes tumor growth and metastasis in gastric cancer. Redox Biol 2018;18:246-255.

Figures. S1 to S3

Figure. S1.


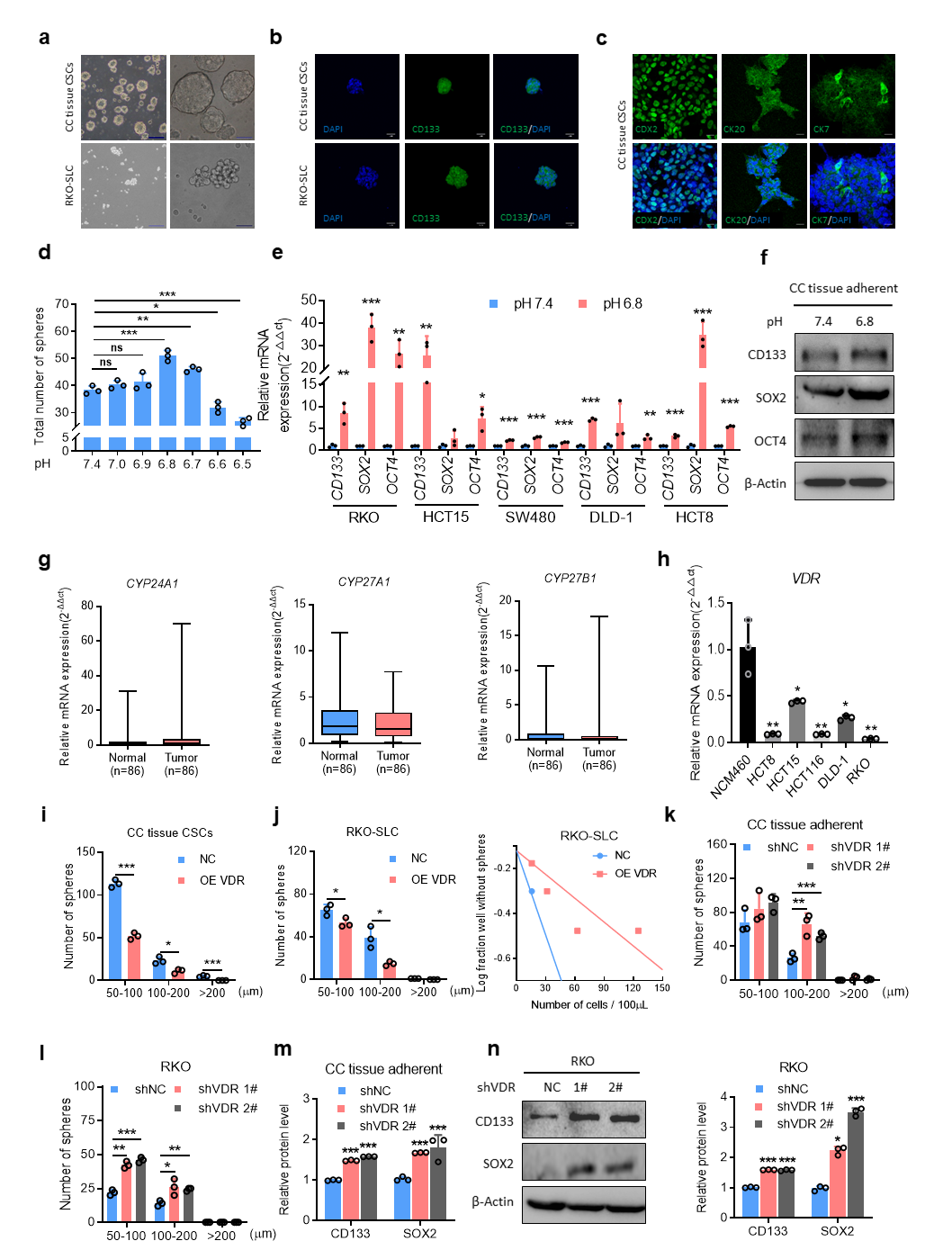


Figure S1. Acidic environment or silencing VDR promoted the stemness of CRC cells.

(a) Representative images of colorectal cancer stem cells (CC tissue CSCs) and RKO colorectal cancer stem-like cells (RKO-SLC) isolated from tissue samples of colorectal cancer patients and RKO cells. Scale bars, 200 μm and 50 μm.

(b) Immunofluorescent staining of CD133 and DAPI in spheres formed by CC tissue CSCs and RKO-SLCs. Scale bars, 50um.

(c) Immunofluorescent staining of CDX2, CK7, CK20 and DAPI in CC tissue CSCs that differentiation was induced through adding fetal bovine serum. Scale bars, 20um.

(d) Tumor sphere formation assay showed the number of spheres (diameters larger than 50 µm) under pH7.4, 7.0, 6.9, 6.8, 6.7, 6.6, 6.5 culture conditions. Student’s t test.

(e) qPCR of *CD133*, *SOX2* and *OCT4* in RKO, HCT15, SW480, DLD1 and HCT8 cells under pH7.4 and pH6.8 conditions.

(f) Immunoblotting of CD133, SOX2 and OCT4 in CC tissue adherent cells cultured under pH 7.4 and pH 6.8.

(g) qPCR of *CYP24A1*, *CYP27A1* and *CYP27B1* in 86 CRC samples and paired adjacent normal samples. Student’s t test.

(h) qPCR of *VDR* in the normal colonic epithelial cell line NCM460 and the HCT8, HCT15, HCT116, DLD1 and RKO cells. Student’s t test.

(i-j) Tumor sphere formation assay showed the size of spheres (diameters larger than 50 µm) of control and VDR-overexpressing CC tissue CSCs and RKO-SLCs (i and j left). And limiting dilution assay of control cells and VDR-overexpressing RKO-SLCs (j right) as described in Fig.2c. Student’s t test.

(k-l) Tumor sphere formation assays showed the size of spheres (diameters larger than 50 µm) formed from cells treated with control or VDR-targeting shRNA as described in Fig.2j. Student’s t test.

(m-n) Immunoblotting of CD133 and SOX2 in RKO cells treated with control or VDR-targeting shRNA (n left). The relative intensity values of images in Fig. 2l and S1n were measured with ImageJ software (m and n right). Student’s t test.

The data are shown as the mean ± SD, **P* < 0.05; ***P* < 0.01; ****P* < 0.001.

Figure. S2.


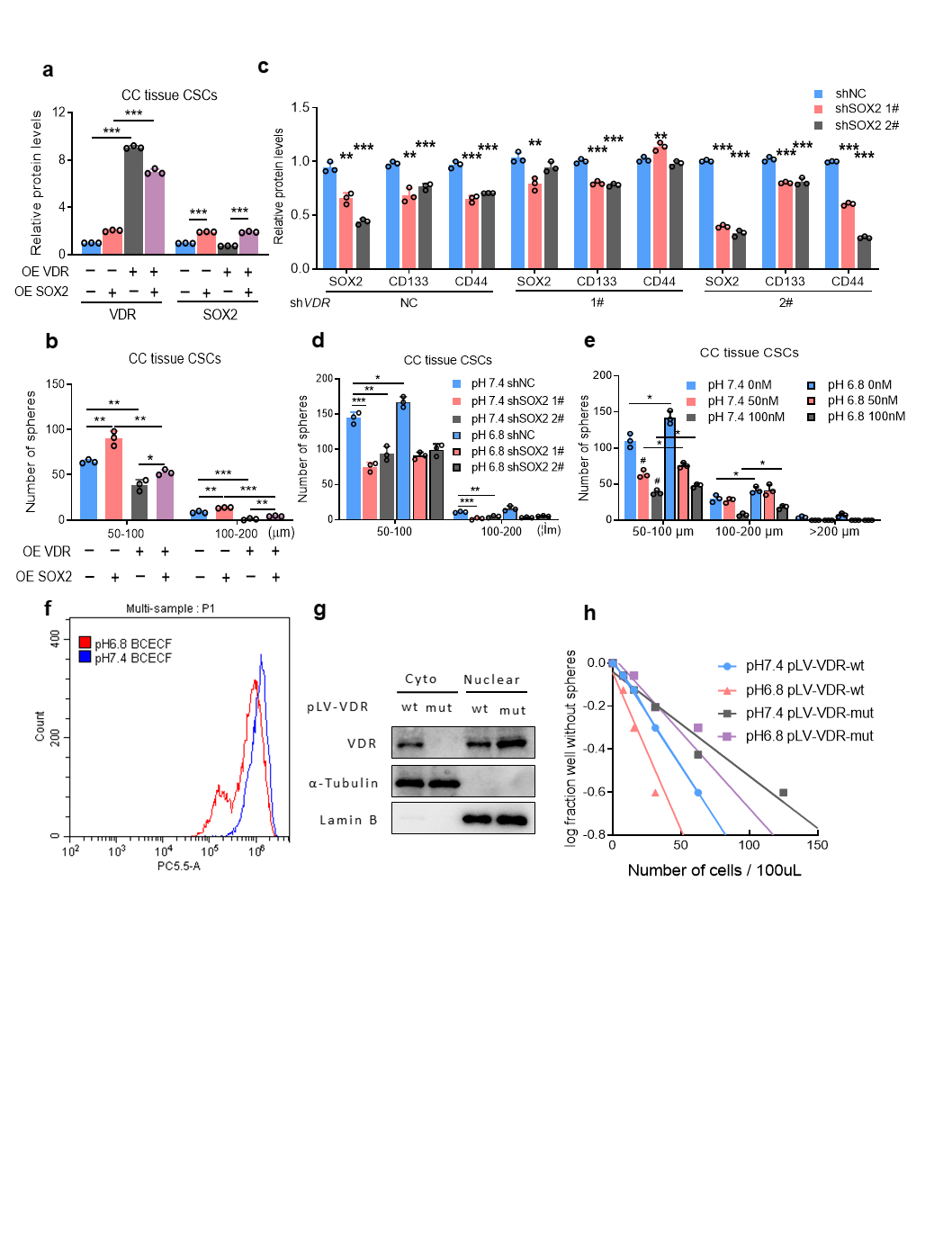


Figure S2. SOX2 could reverse the effects of VDR and PPARD could regulate the expression of VDR.

(a) The relative intensity values of images in Fig. 4a were measured with ImageJ software. Student’s t test.

(b) Tumor sphere formation assay showed the size of spheres (diameters larger than 50 µm) of control and VDR-overexpressing CC tissue CSCs with SOX2 overexpression as described in Fig.4b. Student’s t test.

(c) The relative intensity values of images in Fig. 4c were measured with ImageJ software. Student’s t test.

(d-e) Tumor sphere formation assays showed the size of spheres (diameters larger than 50 µm) formed from control and SOX2 knockdown CC tissue CSCs under pH 7.4 and pH 6.8 conditions (d), CC tissue CSCs that treated with VD_3_ under pH 7.4 and pH 6.8 conditions (e) as described in Fig.4f and 4j. Student’s t test.

(f) The image of BCECF staining in CC tissue adherent cells cultured in pH7.4 and pH6.8 conditions using flow cytometric analysis.

(g) Immunoblotting of VDR in the cytoplasm and nucleus of CC tissue CSCs transfected with pLV-CMV-VDR or pLV-CMV-VDR-mut. α-Tubulin, mainly expressed in cytoplasm. Lamin B, fibrous protein providing structural function and transcriptional regulation in the cell nucleus.

(h) Limiting dilution assay of in CC tissue CSCs transfected with pLV-CMV-VDR or pLV-CMV-VDR-mut under pH 7.4 and pH 6.8 conditions.

The data are shown as the mean ± SD, **P* < 0.05; ***P* < 0.01; ****P* < 0.001.

Figure. S3.


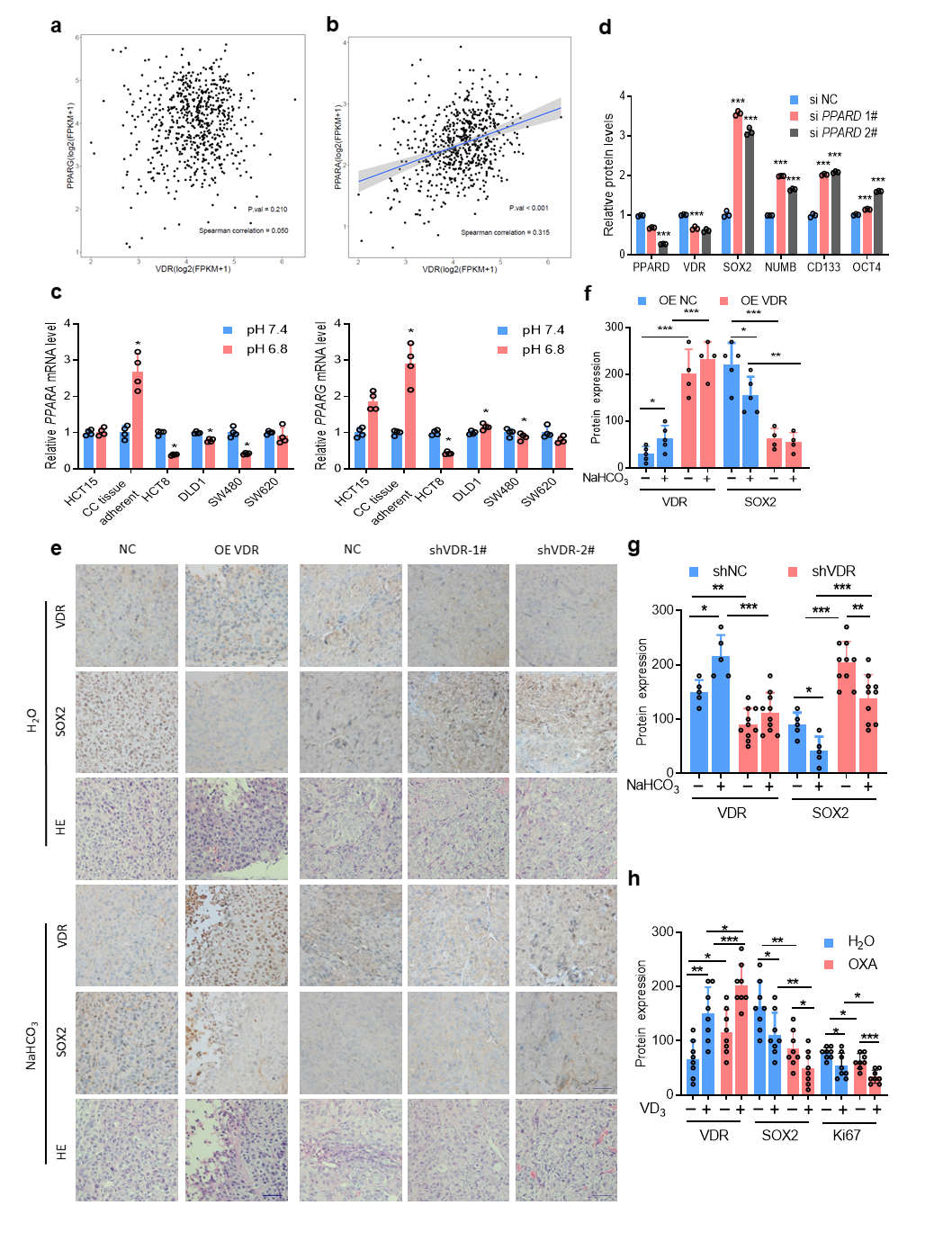


Figure S3. VDR-overexpressing and NaHCO_3_ treatment effectively reduced the expression of SOX2.

(a-b) The correlation between the expression of PPARA and PPARG with VDR in colorectal carcinoma samples from TCGA database.

(c) The relative mRNA expression of PPARA and PPARG in CC tissue adherent, HCT8, DLD1, SW480 and SW620 colorectal cancer cells cultured under pH7.4 and pH6.8. Student’s t test.

(d) The relative intensity values of PPARD, VDR, SOX2, NUMB, CD133 and OCT4 as described in Figure 5l and 5m were measured with ImageJ software. Student’s t test.

(e) Immunohistochemistry showed the VDR and SOX2 expression in xenograft derived from CC tissue CSCs or CC tissue adherent cells as described in Figure 6a and 6b. Scale bars: 50 μm.

(f-g) Quantified data for VDR and SOX2 in CRC tumor tissue samples as described in Figure S3e. Student’s t test.

(h) Quantified data for VDR, SOX2 and Ki67 in CRC tumor tissue samples as described in Figure 6d. Student’s t test.

The data are shown as the mean ± SD, **P* < 0.05; ***P* < 0.01; ****P* < 0.001.

Tables S1 to S2.

Table S1.

| Parameters | Low VDR | High VDR | P |
| --- | --- | --- | --- |
| Age |  |  | 0.303 |
| <57 | 114 (46.3%) | 89 (51.4%) |  |
| ≥57 | 132 (53.7%) | 84 (48.6%) |  |
| Gender |  |  | 0.150 |
| Male | 151 (61.4%) | 94 (54.3%) |  |
| Female | 95 (38.6%) | 79 (45.7%) |  |
| Differentiation |  |  | 0.535 |
| Well/Moderate | 194 (78.9%) | 132 (76.3%) |  |
| Poor | 52 (21.1%) | 41 (23.7%) |  |
| Tumor depth |  |  | 0.402 |
| m/sm/mp(T1/2) | 21 (8.5%) | 19 (11.0%) |  |
| Ss/se/si(T3/4) | 225 (91.5%) | 154 (89.0%) |  |
| Lymph node |  |  | 0.001 |
| Absent | 123 (50.0%) | 58 (33.5%) |  |
| Present | 123 (50.0%) | 115 (66.5%) |  |
| Vascular invasion |  |  | 0.507 |
| Absent | 197 (80.1%) | 143 (82.7%) |  |
| Present | 49 (19.9%) | 30 (17.3%) |  |
| Perineural invasion |  |  | 0.536 |
| Absent | 139 (56.5%) | 103 (59.5%) |  |
| Present | 107 (43.5%) | 70 (40.5%) |  |
| Clinical stage |  |  | 0.006 |
| I-II | 111 (45.1%) | 55 (31.8%) |  |
| III-IV | 136 (54.9%) | 118 (68.2%) |  |

**Table S1 Correlation between VDR expression and clinicopathological features in 419 primary CRC.**

P values determined by Chi-square test using SPSS 20.0. All statistical tests were two-sided.

Table S2.

| **OS** | | | | |
| --- | --- | --- | --- | --- |
| Factors | Univariate | | Multivariate | |
|  | HR (95%CI) | P | HR (95%CI) | P |
| Age |  |  |  | 0.013 |
| (<57/≥57) | 0.64 (0.42, 0.97) | 0.036 | 0.58 (0.37, 0.89) |  |
| Gender |  |  |  | 0.060 |
| (Male/ Female) | 1.83 (1.17, 2.86) | 0.009 | 1.56 (0.98, 2.49) |  |
| Differentiation |  |  |  | 0.251 |
| (Poor/ Well, moderate) | 1.59 (1.01, 2.49) | 0.044 | 1.33 (0.82, 2.14) |  |
| Clinical stage |  | <0.001 |  | 0.036 |
| (III-IV/I-II) | 3.39 (2.00, 5.75) |  | 2.84 (1.07, 7.50) |  |
| Tumor depth |  | 0.251 |  |  |
| (m,sm,mp/ss,se,si) | 0.62 (0.27, 1.41) |  | NA |  |
| Lymph node |  | <0.001 |  | 0.978 |
| (present/absent) | 2.44 (1.53, 3.88) |  | 0.99 (0.43, 2.30) |  |
| Vascular invasion |  | <0.001 |  | 0.002 |
| (abnormal/normal) | 3.27 (2.14, 5.01) |  | 2.14 (1.32, 3.47) |  |
| Perineural invasion |  | 0.002 |  | 0.249 |
| (abnormal/normal) | 1.92 (1.27, 2.90) |  | 1.30 (0.83, 2.03) |  |
| VDR |  | 0.009 |  | 0.004 |
| (high/low) | 0.55 (0.35, 0.86) |  | 0.51 (0.32, 0.81) |  |

**Table S2 Effect of factors on overall survival in CRC patients in the univariate and multivariate cox regression model.**

Hazard ratios and P values were obtained from Cox proportional hazards regression. All statistical test were two-sided.
